# Dexamethasone has profound influence on the energy metabolism of porcine blood leukocytes and prevents the LPS-induced glycolytic switch

**DOI:** 10.3389/fimmu.2025.1514061

**Published:** 2025-02-25

**Authors:** Wenjuan Ma, Julia Brenmoehl, Nares Trakooljul, Klaus Wimmers, Eduard Murani

**Affiliations:** ^1^ Working Group Physiological Genomics, Research Institute for Farm Animal Biology (FBN), Dummerstorf, Germany; ^2^ Working Group Endocrinology of Farm Animals, Research Institute for Farm Animal Biology (FBN), Dummerstorf, Germany

**Keywords:** inflammation, glucocorticoid receptor, immunometabolism, PBMC, dexamethasone, lipopolysaccharide (LPS), GLUT3

## Abstract

In farm animals, little is known about the relationship between energy metabolism of immune cells and their activation state. Moreover, there has recently been evidence that dexamethasone, a powerful glucocorticoid-based drug, can exert its anti-inflammatory effects by interfering with the energy metabolism of immune cells, but the mechanisms are not yet fully understood. To address these knowledge gaps, we explored the connection between the energy metabolism of porcine peripheral blood mononuclear cells (PBMCs) and their response to pro- and anti-inflammatory stimulation with lipopolysaccharide (LPS) and dexamethasone (DEX) *in vitro*. Interventions in the metabolism of PBMCs with the glycolysis inhibitor 2-deoxy-D-glucose or the HIF-1α inhibitor KC7F2 reduced the LPS-induced TNF-α production, but the mitochondrial ATP synthesis inhibitor oligomycin showed no significant effect. The anti-inflammatory action of DEX was not affected by any of the inhibitors. To investigate the metabolic actions of LPS and DEX in PBMCs, we evaluated glycolysis and mitochondrial respiration following 24 hours stimulation using the Seahorse XFe96 flux analyzer. Our results revealed significantly higher glycolysis in LPS-treated PBMCs, but provided no evidence for a change in mitochondrial respiration. In contrast, DEX reduced LPS-induced glycolysis and, especially when administered alone, significantly lowered mitochondrial respiration. Pretreatment with KC7F2 counteracted the effects of LPS and DEX on glycolysis, and reduced mitochondrial respiration regardless of the inflammatory state of the PBMCs. Gene expression analysis identified the glucose transporter *SLC2A3*, and the tricarboxylic acid cycle genes *IDH1* and *SDHB* as the main switches for the antagonistic metabolic actions of LPS and DEX, which are closely associated with the inflammatory state of PBMCs.

## 1 Introduction

Research into the complex, bidirectional interplay between immune and metabolic pathways in immune cells, termed immunometabolism, has gained increasing attention over the last decade. An important driving force is the realization that metabolic pathway choice has a decisive influence on the fate and function of immune cells [reviewed in (1)]. This interest has been reinforced by the introduction of new technologies that have enabled studies of immunometabolism with unprecedented level of detail (2, 3). Resting immune cells have low energy requirements, which are covered mainly by the tricarboxylic acid (TCA) cycle and oxidative phosphorylation (OXPHOS) [reviewed in (1)]. In contrast, activated immune cells have high energy demands and consume high amounts of glucose. A switch to glycolysis not only facilitates rapid production of ATP but also provides intermediate metabolites for the pentose phosphate pathway, which in turn generates precursors for nucleotide and amino acids production supporting cell proliferation, as well as for the generation of reactive oxygen species (ROS) to combat pathogens [reviewed in (4)]. Although the different immune cell types may run distinct metabolic programs when activated, enhanced glycolysis has been observed in many of them, including M1 macrophages and Th17 lymphocytes [reviewed in (4)]. In fact, elevated glycolysis is considered a hallmark of pro-inflammatory cells (5). Anti-inflammatory cells, on the other hand, gain energy mainly from OXPHOS and fatty acid oxidation [reviewed in (4)]. We are just beginning to understand the factors that modulate metabolism of immune cells and ultimately determine their immunophenotype [reviewed in (1)]. While this research is very active in animal models (6–10) and humans (2, 11–14), there is little knowledge about immunometabolism in farm animals (15), despite the discussion about possible trade-offs between growth performance and the immune response due to shared pathways targeted by selective breeding for increased lean growth (16). It is important to note that although some fundamental mechanisms appear to be conserved between species, there are also notable differences. For instance, whereas both mouse and human macrophages induce glycolysis upon inflammatory activation, mitochondrial respiration is decreased only in mouse macrophages (2). This illustrates that findings cannot be readily transferred between species, making research on farm animals imperative.

Previously, we investigated the effect of a natural gain-of-function substitution in the porcine glucocorticoid receptor (GR), referred to as GR_Ala610Val_, on the function of porcine peripheral (blood mononuclear cells) blood mononuclear cells (PBMCs) (17). One of the most remarkable findings was that the substitution influences various metabolic pathways both in resting PBMCs as well as after stimulation with lipopolysaccharide (LPS, activating inflammatory reaction) and dexamethasone (DEX, an anti-inflammatory drug and a selective ligand activating GR). Glucocorticoid receptor exerts a strong influence both on immune cell function as well as on metabolism (18). Yet, its role in the immunometabolic interplay has only recently been recognized (19, 20) and is still poorly understood. In order to shed new light on immunometabolism in pigs and on the role of GR signaling in this process, in the present study we investigated metabolic responses of porcine PBMCs to stimulation with LPS, DEX, and their combination in relation to the immune response. We included PBMCs from different housing conditions to examine if these impact the metabolic response of PBMCs to stimulation. Because PBMCs can be obtained in routine practice for diagnostic purposes, they are particularly suitable as surrogate cells to explore the potential of different interventions to influence immune cell function. Finally, we explored the underlying mechanisms via pharmacological modulation of hypoxia-inducible factor 1 alpha (HIF-1α), a key factor rewiring metabolism of immune cells depending on their inflammatory status (21), and via transcriptome analyses of key genes influencing immunometabolism.

## 2 Materials and methods

### 2.1 Animals

All blood samples used to isolate PBMCs were collected from purebred German Landrace pigs raised at the experimental pig farm (EAS) of the Research Institute for Farm Animal Biology (FBN). The blood samples used to make PBMC pools were obtained from conventionally-reared slaughter pigs (~160 days old), sampled as part of a previous study (17). A total of four pools representing in total 34 pigs of both sexes were used. In addition, individual PBMC samples were obtained from 160 days old pigs reared in conventional conditions (designated TW1 according to German animal husbandry welfare label; n=14 balanced for sex) and from 160 days old pigs reared in enriched conditions (designated TW3 according to German animal husbandry welfare label; n=10 balanced for sex) (for more information on the husbandry conditions please see **Supplementary Material**). The Animal Care Committee of the FBN and the State Mecklenburg-Western Pomerania (Landesamt für Landwirtschaft, Lebensmittelsicherheit und Fischerei; LALLF) approved the animal experiment (Approval number 7221.3-1-029/21).

### 2.2 Isolation of porcine PBMCs

Approximately 50 mL of trunk blood was collected from each pig during exsanguination using 50 mL conical tubes containing 1 mL of 0.5 M EDTA to prevent blood coagulation, and put on wet ice. Porcine PBMCs were isolated through a density gradient centrifugation method using Histopaque-1077 (Sigma-Aldrich, Taufkirchen, Germany) and cryopreserved as previously described (22).

### 2.3 Cell culture and treatment

To explore the influence of PBMC metabolism on response to stimulation, the pooled PBMC samples (n=4, set-up as mentioned above, for each combination of pretreatment and treatment) were seeded in 96-well cell culture plates (Faust Lab Science, Klettgau, Germany) at a density of 2.5 × 10^5^ cells per well in standard complete medium consisting of RPMI1640 medium (PAN Biotech, Aidenbach, Germany) supplemented with 10% fetal calf serum (PAN Biotech), 2 mM L-glutamine (PAN Biotech), 100 U/mL penicillin/100 µg/mL streptomycin (PAN Biotech), and cultured in a 37°C, 5% CO_2_ environment overnight. The next day, the cells were pretreated for 30 minutes with increasing concentrations of either oligomycin (ThermoFisher, Darmstadt, Germany) - a mitochondrial ATP synthesis inhibitor (0, 1.25, 2.5, 5, and 10 µM), 2-deoxy-D-glucose (2-DG) (ThermoFisher) - a glycolysis inhibitor (0, 0.625, 1.25, 2.5, and 5 mM), or KC7F2 (Bio-Techne, Wiesbaden-Nordenstadt, Germany) - a HIF-1α inhibitor (0, 6.25, 12.5, 25, and 50 µM). Following this, the cells were stimulated for 24 hours with either vehicle, 100 ng/mL LPS (Sigma, Escherichia coli O111: B4 serotype), 100 nM DEX (Sigma), or a combination of both. Finally, after the stimulation, the supernatant was carefully collected by centrifugation at 515 g for 7 minutes and used for bioassays as described below.

For metabolic flux analyses, individual PBMC samples were resuscitated in standard complete medium at 37°C, 5% CO_2_ environment overnight. Subsequently, the cells were harvested and reseeded onto a Seahorse XFe96 PDL cell culture microplate (Agilent, Waldbronn, Germany) at 2 × 10^5^ cells/well (the cell number was determined in preliminary experiments as recommended by the manufacturer using either 0.5 × 10^5^, 1 × 10^5^, 1.5 × 10^5^, or 2 × 10^5^ PBMCs/well) in standard complete medium, and cultured in a 37°C, 5% CO_2_ environment. Before bioenergetics analysis, the cells were challenged using the same stimulants and concentrations as described above. Details of the metabolic flux analyses are described in section 2.5.

The *in vitro* experiment for gene expression analysis was performed using 3 × 10^6^ cells/well from each of the pooled PBMC samples in 6-well cell culture plates (Faust Lab Science), essentially as described above. After overnight incubation, the cells received no pretreatment or were pretreated with 12.5 µM of KC7F2 for 30 minutes. Subsequently, the non-pretreated cells were stimulated for 24 hours with either vehicle, 100 ng/mL LPS, 100 nM DEX, or a combination of 100 ng/mL LPS and 100 nM DEX. The pretreated cells were stimulated using either 100 ng/mL LPS alone or in combination with 100 nM DEX. After stimulation, the cells were harvested, lysed in TRI-Reagent (Sigma), and stored at -80°C until further processing for RNA extraction. Transcriptional response of selected genes to treatment was measured as described in section 2.6.

### 2.4 Bioassays

Since the modulators of cell metabolism can influence cell vitality, cytotoxicity assays were performed by measuring extracellular lactate dehydrogenase (LDH) concentration in the collected supernatants using the colorimetric CyQUANT LDH Cytotoxicity Assay (ThermoFisher). The concentration of TNF-α in the supernatant was assessed using a widely used (23) porcine ELISA (Bio-Techne). Both assays were performed as per manufacturer protocol, and the resulting absorbances were measured using a FLUOstar Omega microplate reader (BMG LABTECH, Ortenberg, Germany).

### 2.5 Metabolic flux analyses

After 24 hours of PBMC stimulation, as described in section 2.3, the metabolic flux was measured on a Seahorse XFe96 instrument (Agilent). The cells were washed twice with either Mito Stress Test Assay Medium (XF RPMI medium, pH 7.4, supplemented with 1 mM pyruvate, 2 mM glutamine, and 10 mM glucose) or Glycolysis Stress Test Assay Medium (XF RPMI medium, pH 7.4, supplemented with 2 mM glutamine), the final volume (180 µL) of corresponding assay medium was added, and cells were incubated at 37°C without CO_2_ for 45 minutes to allow equilibration. Mitochondrial and glycolytic activity were assayed using the Mito Stress Test kit and Glycolysis Stress Test kit, respectively, according to manufacturer instructions (Agilent). Briefly, mitochondrial respiration function was assessed by measuring the oxygen consumption rate (OCR) after consecutively injecting oligomycin (1 µM), FCCP (an electron transport chain accelerator, 2 µM), and antimycin A (a complex III inhibitor, 0.5 µM) along with rotenone (a complex I inhibitor, 0.5 µM). The glycolytic rate was quantified by measuring the extracellular acidification rate (ECAR) after the injections of glucose (10 mM), followed by oligomycin (2 µM), and finally, 2-DG (50 mM). The concentration of the injected compounds was optimized along with cell density in preliminary experiments. Data were normalized to protein content per well obtained using Pierce BCA Protein Assay Kit (ThermoFisher) (13).

### 2.6 Quantification of gene expression

Expression of selected genes was measured using quantitative real-time PCR (qPCR) as described previously (24). Briefly, aqueous phase was obtained from cells lysed in TRI-Reagent, and total RNA was purified (including on-column DNase digestion) with the help of the RNA Clean&Concentrator-5 Kit (Zymo Research, Freiburg, Germany). Reverse transcription was performed in a reaction containing 500 ng total RNA, 500 ng random hexamers (Promega, Mannheim, Germany), 500 ng of oligo d(T)13 VN, 40 units of RNasin Plus (Promega), and 200 units of SuperScript III reverse transcriptase (ThermoFisher). The qPCR was carried out using the LightCycler 480 SYBRplus Green I Master kit on a LightCycler 480 System (Roche, Mannheim, Germany). For absolute quantification, a standard curve based on a serial dilution of a gene-specific PCR fragment was generated for each target. The results were normalized using *EXOC1* as internal reference gene. Information on primers used for qPCR is summarized in **Supplementary Table S1**.

### 2.7 Data analysis

Statistical analysis was performed using GraphPad Prism 9.5.1 (GraphPad Software, San Diego, CA, USA). Statistical tests with a p < 0.05 are reported as significant. The influence of different pretreatments on the response to stimulation (TNF-α concentration) or on metabolic flux parameters was analyzed using two-way ANOVA. The differences between means were tested using the Tukey´s multiple comparison test or the Dunnett´s multiple comparison test (for metabolic flux), respectively. The effects of treatment, sex, and husbandry conditions on metabolic flux in individual samples were tested using a mixed-effects model and the Tukey´s test. Gene expression data obtained by qPCR were log2 transformed before analysis. The transcriptional response to different treatments was analyzed by one-way ANOVA. Preplanned comparisons were performed using the Šidák´s test. Principal component and correlation analysis was performed on the log2 transformed gene expression matrix. Whole transcriptome data were obtained from our previous study (accession number E-MTAB-9808) and re-analyzed based on the Ensemble Gene version 111 as described previously (25).

## 3 Results

### 3.1 Immune response of porcine PBMCs is influenced by their energy metabolism

**Figure 1:**
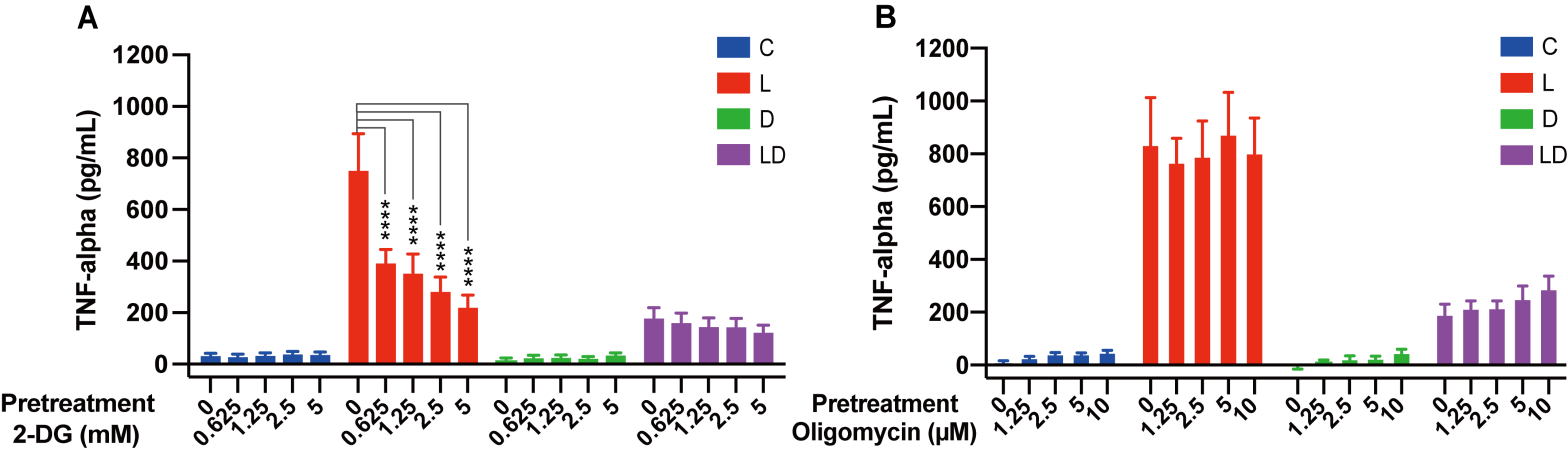
The glycolysis inhibitor 2-DG, but not the mitochondrial ATP synthase inhibitor oligomycin, influence LPS-induced TNF-α secretion in PBMCs. Four pooled PBMC samples were pretreated with various concentrations of **(A)** 2-DG (0, 0.625, 1.25, 2.5, and 5 mM) or **(B)** oligomycin (0, 1.25, 2.5, 5, and 10 μM) for 30 minutes, followed by stimulation with vehicle control (labeled by C, blue), 100 ng/mL LPS (L, red), 100 nM DEX (D, green), or a combination of LPS and DEX (LD, purple) for 24 hours. TNF-α secretion was measured using ELISA. Statistical analysis was performed using two-way ANOVA, followed by Dunnett's multiple comparison test. The bars show mean + standard error of the mean. ****p < 0.0001.

To examine whether there is an interaction between energy metabolism and immune phenotype of porcine PBMCs, we pretreated the cells *in vitro* with increasing concentrations of 2-DG, a glycolysis inhibitor, or oligomycin, a mitochondrial ATP synthesis inhibitor, before stimulating the pro- and anti-inflammatory responses by LPS and/or DEX. To reduce the typically considerable individual variation in responses to immune challenges, and to have sufficient material for follow-up experiments, we used pooled PBMC samples. The immune response was monitored based on the concentration of TNF-α, a master regulator of pro-inflammatory cytokines whose secretion is influenced by the glycolytic pathway (26). As expected, there was a significant effect of treatment on TNF-α concentration (p < 0.0001), which increased above the low level of untreated cells (C, **Figures 1A, B**) only in the presence of LPS (LPS and LPS+DEX, **Figures 1A, B**) (for an overview of the statistical analysis results, see **Supplementary Table S2**). The stimulatory effect of LPS was significantly reduced by co-treatment with DEX (LPS+DEX, **Figures 1A, B**). Pretreatment with 2-DG dose-dependently reduced the LPS-induced TNF-α production, but had no significant influence on the anti-inflammatory effect of DEX in the LPS+DEX treatment (**Figure 1A**; **Supplementary Table S2A**). In contrast, pretreatment with oligomycin showed no statistically significant influence on the effect of either LPS or DEX on TNF-α production (**Figure 1B**; **Supplementary Table S2B**). These results confirmed that the pro-inflammatory response is connected to the glycolytic pathway in porcine PBMCs, similar to humans and rodents. On the other hand, inhibition of mitochondrial ATP production does not appear to have a noticeable influence on the inflammatory state of the porcine PBMCs.

### 3.2 LPS and DEX modulate the metabolic flux of porcine PBMCs in opposite directions

To further characterize the interaction between immune and metabolic activity in PBMCs, we examined their bioenergetics following LPS and/or DEX treatment *in vitro* using the Seahorse extracellular flux analyzer. For these experiments, we utilized individual samples, which also allowed us to study the impact of sex and husbandry conditions on the immunometabolic phenotype of porcine PBMCs.

**Figure 2:**
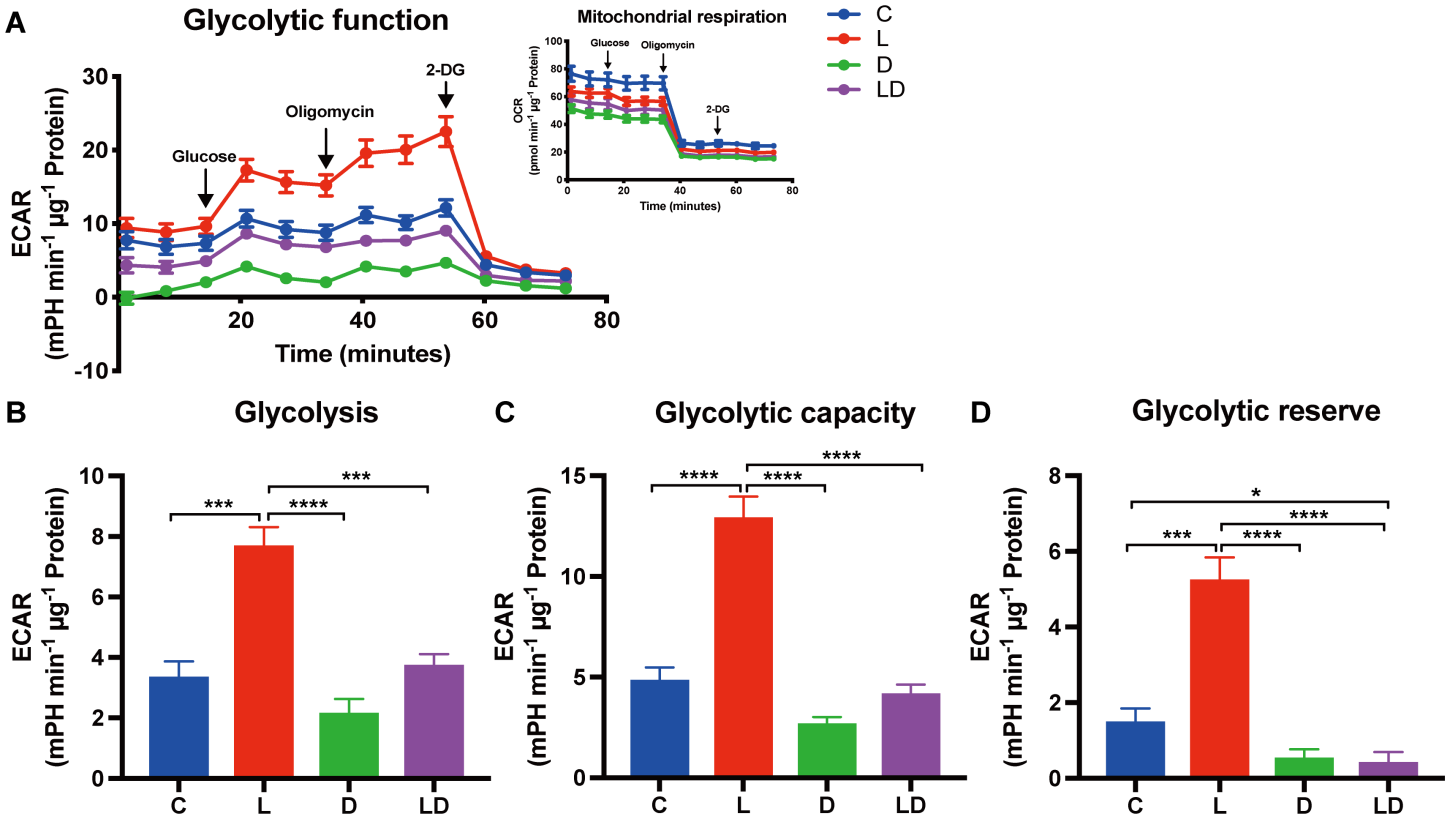
LPS and DEX show opposite effects on the glycolytic switch induced by LPS in PBMCs. The glycolytic metabolism of PBMCs depending on the inflammatory status was examined using the Glycolysis Stress Test on a Seahorse XFe96 analyzer. PBMCs from 24 individual samples from two different environments were treated with vehicle control (labeled by C, blue), 100 ng/mL LPS (L, red), 100 nM DEX (D, green), or a combination of LPS and DEX (LD, purple) for 24 hours prior to the test. The data were normalized to protein content per well and analyzed using Agilent WAVE software. **(A)** The extracellular acidification rate (ECAR) and oxygen consumption rate (OCR, inset) were recorded before and after the sequential addition of glucose (10 mM), oligomycin (2 µM), and 2-DG (50 mM). **(B)** Glycolysis, **(C)** Glycolytic capacity, and **(D)** Glycolytic reserve were calculated. ECAR data were analyzed using two-way ANOVA, followed by Tukey’s multiple comparison test. The results are presented as mean + standard error of the mean. *p < 0.05, ***p < 0.001, and ****p < 0.0001.

To investigate the influence of LPS and DEX on glycolytic metabolism, we conducted the Glycolysis Stress Test. **Figure 2** displays real-time curves of extracellular acidification rate (ECAR) and oxygen consumption rate (OCR) (inset) from the test (**Figure 2A**), as well as the derived glycolytic parameters (**Figures 2B–D**), depending on the treatment. There was a significant treatment effect (p < 0.0001), whereby stimulation with LPS alone significantly enhanced glycolysis, glycolytic capacity as well as glycolytic reserve compared to control (**Figures 2B–D**). Co-treatment with DEX significantly mitigated the LPS-induced increase in all glycolytic parameters and reduced them to the level of untreated cells (**Figures 2B, C**) or even below (**Figure 2D**). Moreover, cells treated with DEX alone tended to show the lowest glycolytic parameters (**Figures 2B–D**). We found no statistically significant effect of sex or husbandry conditions on the outcome of the Glycolysis Stress Test (**Supplementary Table S2C**).

**Figure 3:**
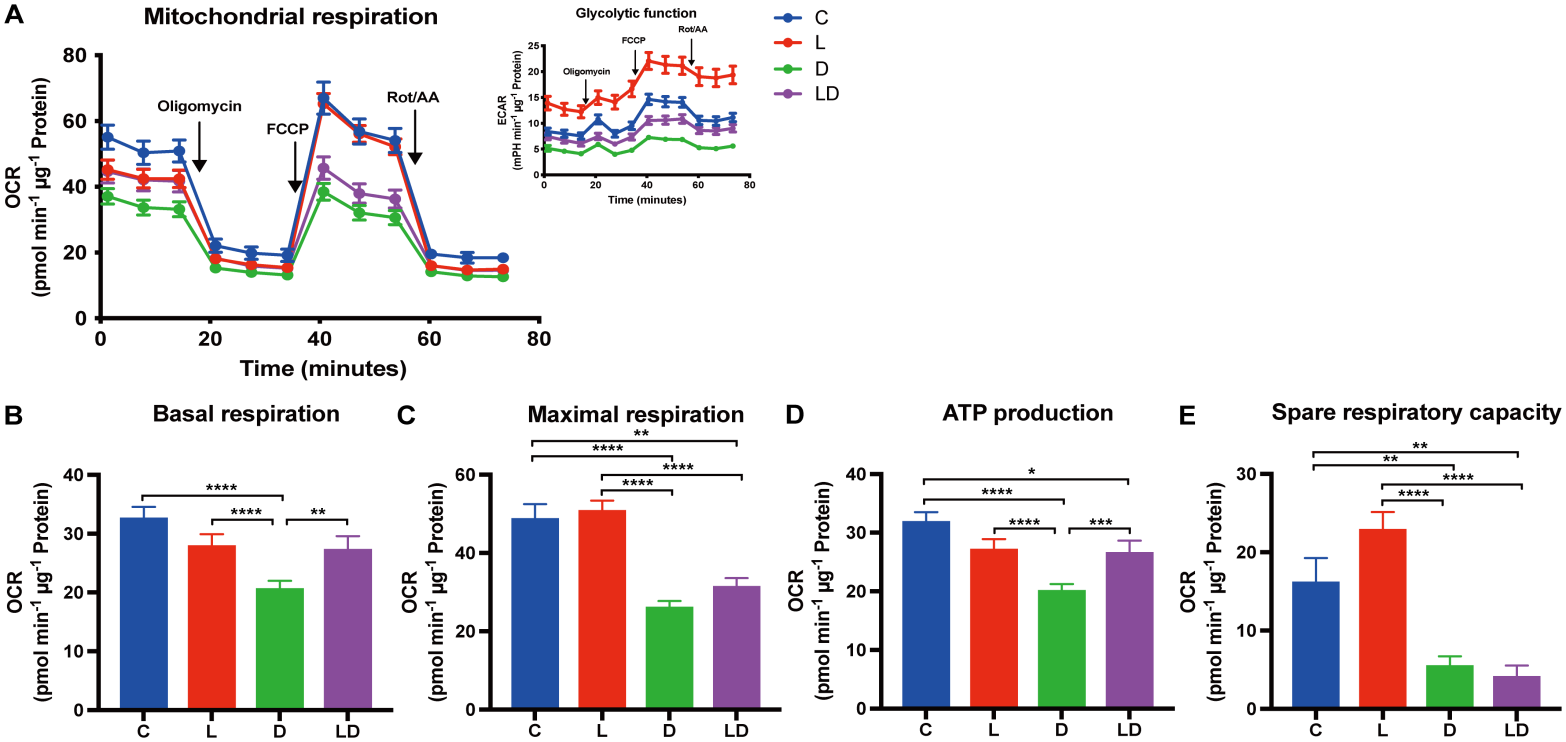
DEX reduces mitochondrial respiration in PBMCs. Mitochondrial respiration of PBMCs depending on the inflammatory status was examined using the Mito Stress Test on a Seahorse XFe96 analyzer. PBMCs from 24 individual samples from two different environments were treated with vehicle control (labeled by C, blue), 100 ng/mL LPS (L, red), 100 nM DEX (D, green), or a combination of LPS and DEX (LD, purple) for 24 hours prior to the test. The data were normalized to protein content per well and analyzed using Agilent WAVE software. **(A)** The oxygen consumption rate (OCR) and extracellular acidification rate (ECAR, inset) were recorded before and after the addition of oligomycin (1 µM), FCCP (2 µM), and rotenone/antimycin A (Rot/AA) (0.5 µM). **(B)** Basal respiration, **(C)** Maximal respiration, **(D)** ATP production, and **(E)** Spare respiratory capacity were calculated. OCR data were analyzed using two-way ANOVA, followed by Tukey’s multiple comparison test. The results are presented as mean + standard error of the mean. *p < 0.05, **p < 0.01, ***p < 0.001, and ****p < 0.0001.

Next, we examined how mitochondrial respiration is altered by LPS and DEX using the Mito Stress Test. The results are summarized in a similar way to the Glycolysis Stress Test in **Figure 3**. We found a statistically significant effect of treatment (p < 0.0001) on basal and maximal respiration, ATP production, and spare respiratory capacity (**Figures 3B–E**). Sex or husbandry conditions showed no statistically significant effects (**Supplementary Table S2D**). LPS stimulation caused a 1.4-fold increase in spare respiratory capacity compared to the control condition (**Figure 3E**), but neither this nor any other parameter showed a significant difference after LPS treatment compared to control (**Figures 3B–E**). In contrast, treatment with DEX alone significantly decreased all parameters of mitochondrial respiration compared to control, and also significantly reduced maximal respiration and spare respiratory capacity when co-applied with LPS compared to LPS applied alone (**Figures 3C, E**).

Taking the results of both tests together, LPS and DEX influence the metabolism of porcine PBMCs in opposite directions – LPS shifts towards enhanced glycolysis, and DEX induces an overall low energetic state (**Supplementary Material**, **Supplementary Figure 1**).

### 3.3 The HIF-1α inhibitor KC7F2 reduces inflammatory response to LPS and alters the metabolic effects of LPS and DEX

As a next step, we investigated whether there is a connection between the function of HIF-1α and immune and metabolic actions of LPS and DEX in porcine PBMCs. HIF-1α is a transcriptional factor that plays a central role in immunometabolism, primarily by promoting glycolysis and enhancing interleukin 1β production (21). Furthermore, it has recently been suggested that the anti-inflammatory effect of glucocorticoids (DEX) in murine and human macrophages involves downregulation of HIF-1α mRNA and/or protein abundance, and thus counteracting its metabolic actions (19, 20).

**Figure 4:**
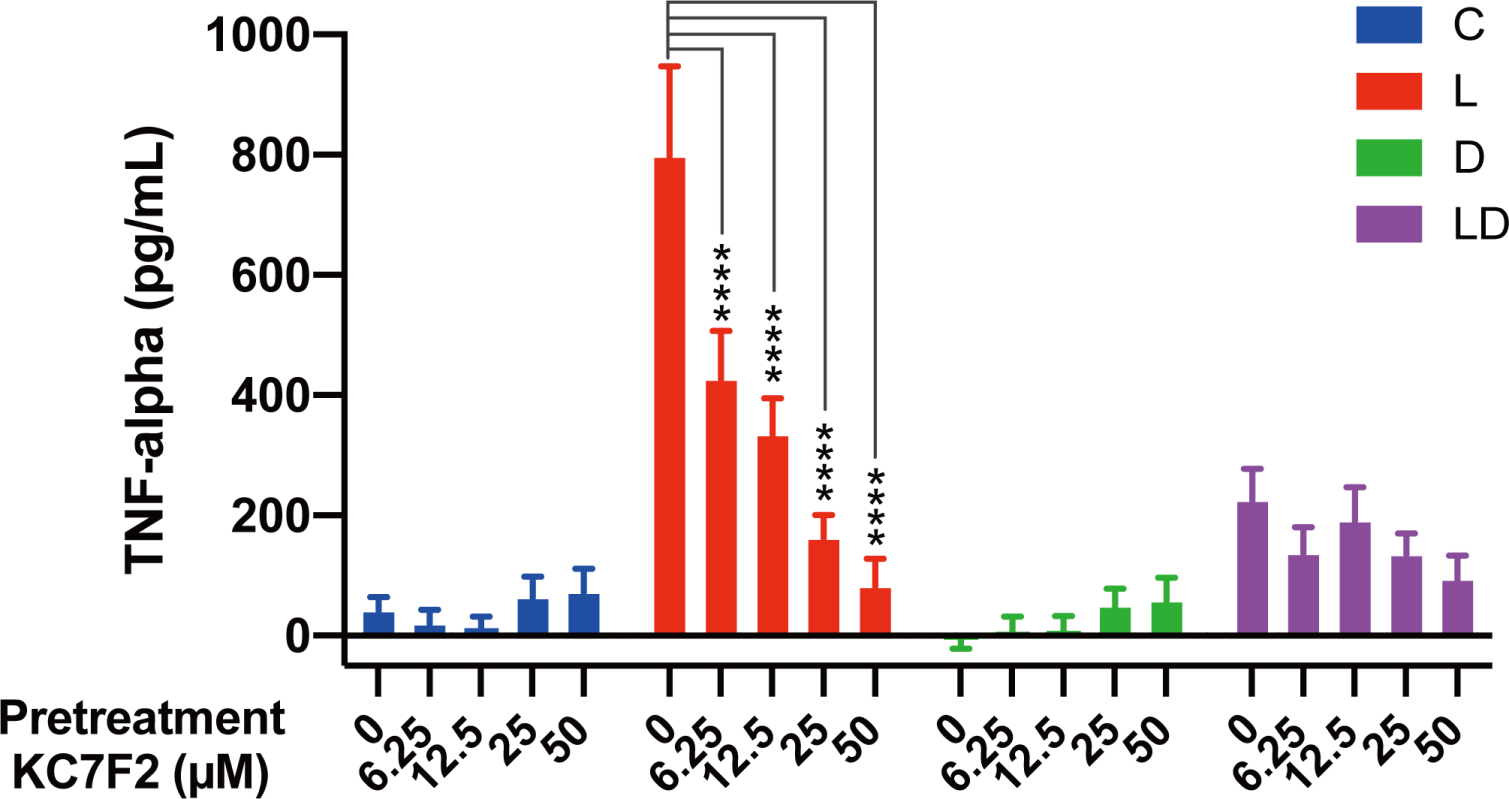
The HIF-1α inhibitor KC7F2 reduces LPS-induced TNF-α secretion in PBMCs. Four pooled PBMC samples were pretreated with varying concentrations of KC7F2 (0, 6.25, 12.5, 25, and 50 µM) for 30 minutes, followed by stimulation with vehicle control (labeled by C, blue), 100 ng/mL LPS (L, red), 100 nM DEX (D, green), or a combination of LPS and DEX (LD, purple) for 24 hours. TNF-α secretion was measured using ELISA. Statistical analysis was conducted using two-way ANOVA followed by Dunnett’s multiple comparison test. The results are presented as mean + standard error of the mean. ****p < 0.0001.

To examine the role of HIF-1α in the regulation of the inflammatory state of porcine PBMCs by LPS and DEX, we performed a similar experiment as described above for 2-DG and oligomycin, but this time the cells were pretreated with increasing doses of KC7F2, a HIF-1α inhibitor that downregulates HIF-1α protein synthesis (20). The application of KC7F2 showed a similar influence on TNF-α concentration as 2-DG: the LPS-induced TNF-α production was dose-dependently lowered by KC7F2, but the inhibitory effect of DEX was not significantly affected (**Figure 4**; **Supplementary Table S2E**).

**Figure 5:**
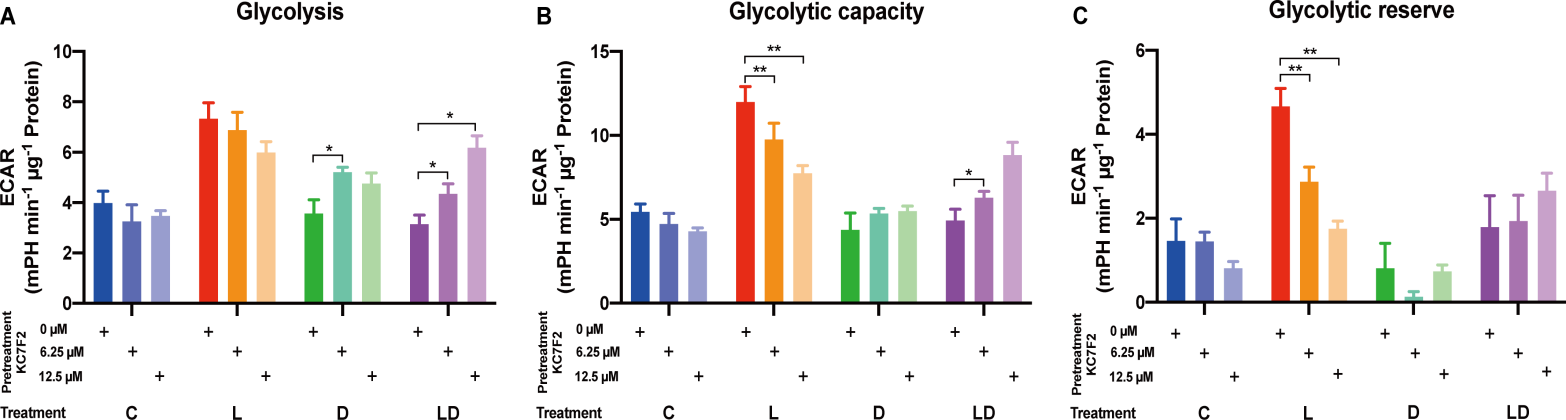
The HIF-1α inhibitor KC7F2 counteracts the effects of LPS and DEX on glycolysis in PBMCs. The impact of KC7F2 on glycolysis depending on the inflammatory status was examined using the Glycolysis Stress Test on a Seahorse XFe96 analyzer. Four pooled PBMC samples were pretreated with KC7F2 (0, 6.25, or 12.5 µM) for 30 minutes, followed by stimulation with either vehicle control (labeled by C, blue), 100 ng/mL LPS (L, red), 100 nM DEX (D, green), or a combination of LPS and DEX (LD, purple) for 24 hours. Data were normalized to protein content per well and analyzed using Agilent WAVE software, yielding: **(A)** Glycolysis, **(B)** Glycolytic capacity, and **(C)** Glycolytic reserve. ECAR data were analyzed by two-way ANOVA followed by Dunnett’s multiple comparison test. The results are presented as mean + standard error of the mean. *p < 0.05, **p < 0.01.

**Figure 6:**
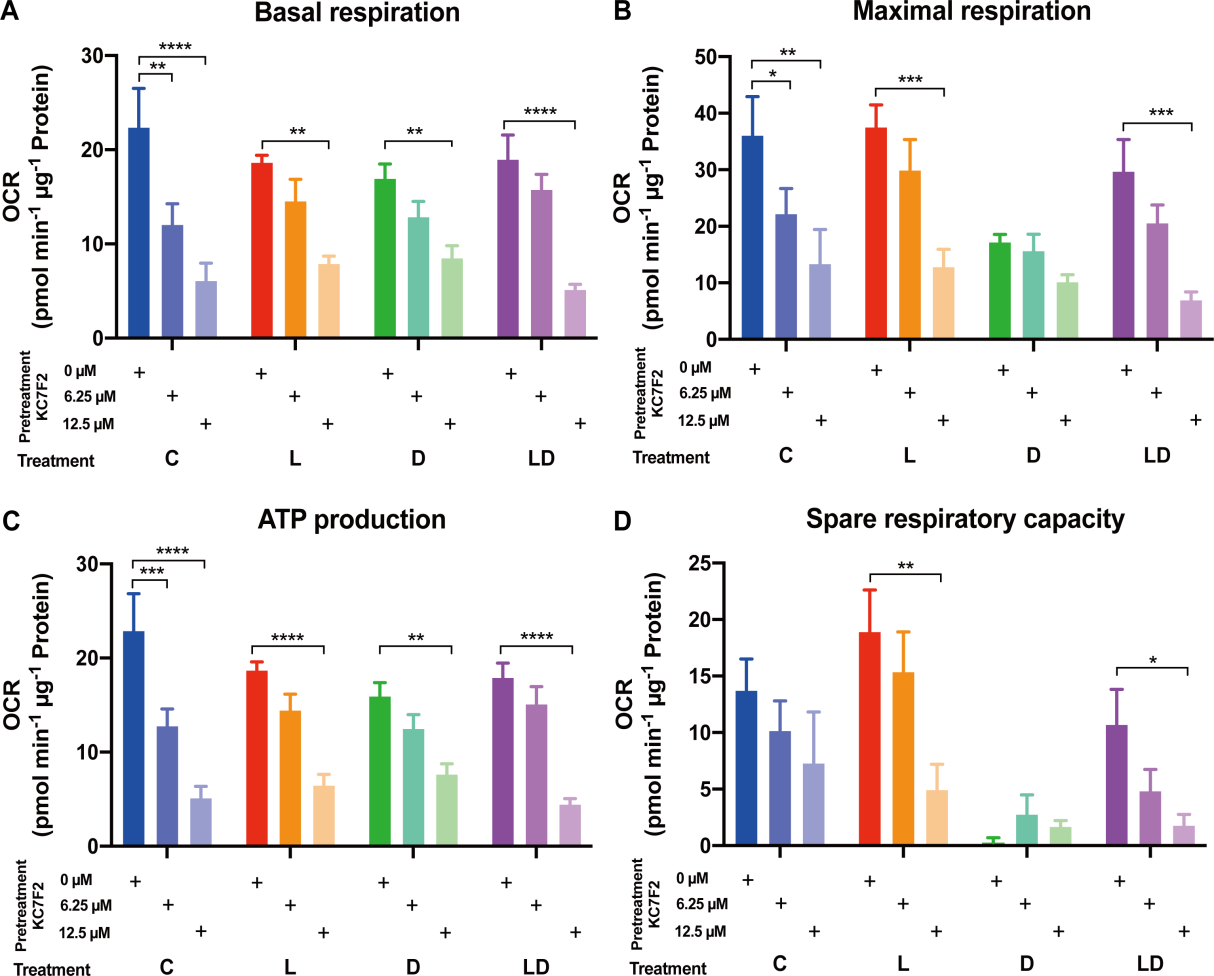
The HIF-1α inhibitor KC7F2 reduces mitochondrial respiration in PBMCs regardless of the inflammatory status. The impact of KC7F2 on mitochondrial respiration depending on the inflammatory status was examined using the Mito Stress Test on a Seahorse XFe96 analyzer. Four pooled PBMC samples were pretreated with KC7F2 (0, 6.25, or 12.5 µM) for 30 minutes, followed by stimulation with vehicle control (labeled by C, blue), 100 ng/mL LPS (L, red), 100 nM DEX (D, green), or a combination of LPS and DEX (LD, purple) for 24 hours. Data were normalized to protein content per well and analyzed using Agilent WAVE software, yielding: **(A)** Basal respiration, **(B)** Maximal respiration, **(C)** ATP production, and **(D)** Spare respiratory capacity. OCR data were analyzed by two-way ANOVA followed by Dunnett’s multiple comparison test. The results are presented as mean + standard error of the mean. *p < 0.05, **p < 0.01, ***p < 0.001, and ****p < 0.0001.

Having established the involvement of HIF-1α in the LPS response, we set out to investigate its role in the metabolic rewiring of porcine PBMCs by LPS and DEX. To this end, we examined the impact of KC7F2 on the bioenergetic effects of LPS and/or DEX treatment in the pooled PBMC samples. We applied the two lower KC7F2 doses (6.25 and 12.5 µM) because the higher doses showed a negative impact on cell vitality as indicated by the LDH cytotoxicity assay (**Supplementary Material**, **Supplementary Figure 2**), and as also reported by Clayton et al. (20). Without KC7F2, both the Glycolysis and Mito Stress Tests (**Figures 5**, **6**; **Supplementary Tables S2F, G**) showed essentially the same effects of LPS and DEX as before in individual samples (**Figures 2**, **3**), demonstrating the robustness of our findings. In the Glycolysis Stress Test, KC7F2 exhibited a treatment-dependent effect. The LPS-induced glycolytic shift was dose-dependently attenuated by KC7F2 addition, with a statistically significant reduction in glycolytic capacity and reserve (**Figures 5A–C**). Surprisingly, in the presence of DEX, KC7F2 tended to increase the glycolytic parameters, particularly in the combined LPS+DEX treatment, with statistically significant increases in glycolysis and glycolytic capacity (**Figures 5A, B**). Mitochondrial respiration, in turn, was consistently reduced by KC7F2 in a dose-dependent manner across treatments, with the 12.5 µM dose significantly reducing essentially all parameters regardless of treatment (**Figures 6A–C**). Our results show that in the LPS-induced pro-inflammatory state, KC7F2 has similar effects on the metabolism of PBMCs as DEX. In contrast, KC7F2 appears to counteract the suppressing effect of DEX on glycolysis, but its suppressing effect on mitochondrial respiration is independent of the treatment, i.e., the immune state. These observations thus support the hypothesis that the metabolic action of DEX in porcine PBMCs may be determined by an interaction between GR and HIF-1α signaling.

### 3.4 The anti-inflammatory and metabolic actions of DEX are counteracted by the HIF-1α inhibitor KC7F2

In order to gain insight into the mechanisms underlying the antagonistic action of LPS and DEX on the immunometabolic phenotype of porcine PBMCs, we re-analyzed our previously published whole transcriptome data (25) with particular focus on metabolic genes. Specifically, we extracted genes belonging to the glycolysis, TCA, and OXPHOS KEGG pathways to infer the direction of their regulation by LPS and DEX using Ingenuity Pathway Analysis (IPA). The analysis revealed changes in specific pivotal genes (e.g. *PFKFB3*, **Supplementary Table S3**), but the z-scores were rather low and lacked a clear trend (**Supplementary Table S4**). It is important to note that in the previous *in vitro* experiment we used different conditions, particularly an early time point (2 hours), as well as different LPS and DEX doses, which may have contributed to this.

**Figure 7:**
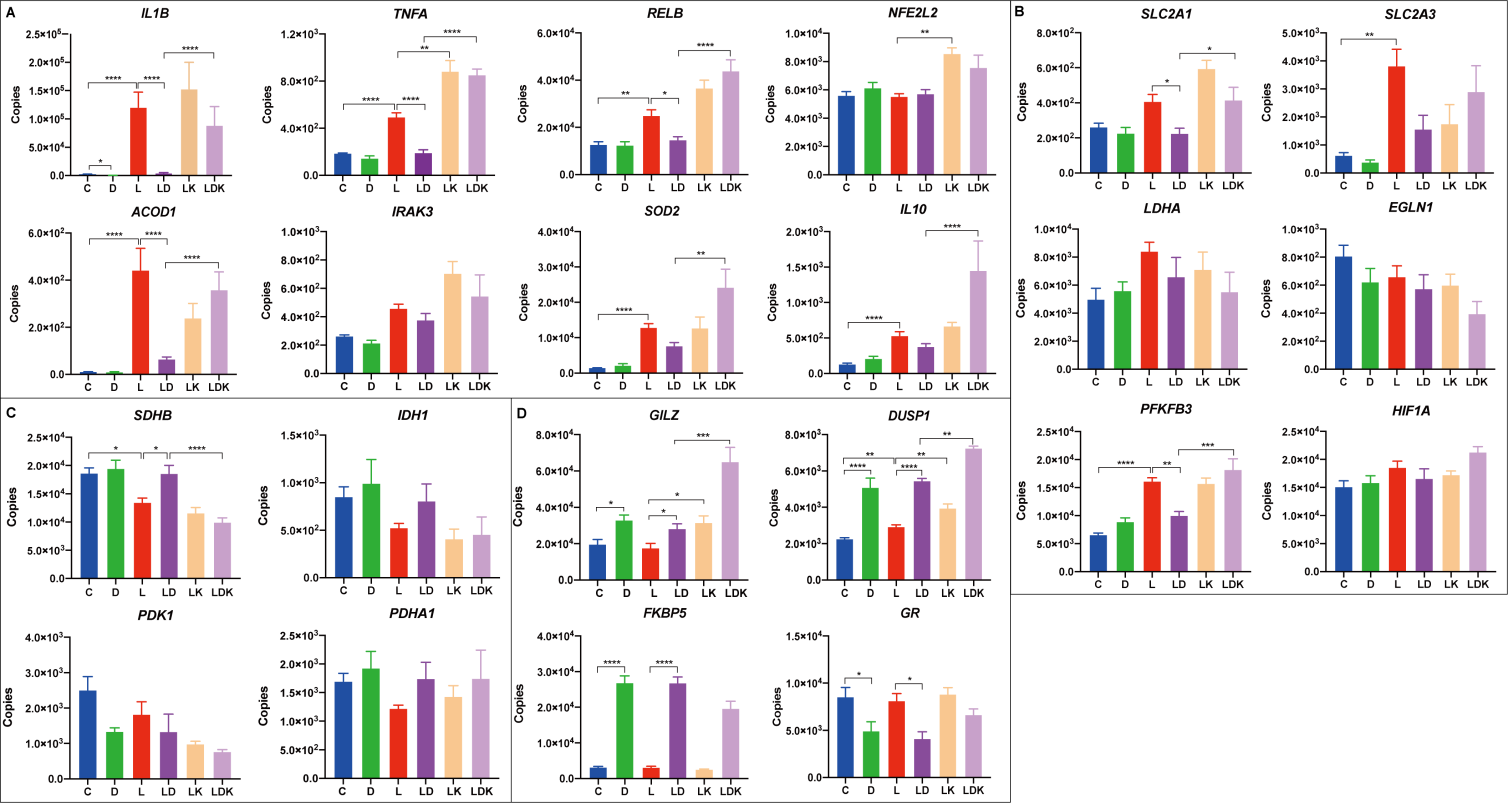
Transcriptional responses to LPS and DEX are influenced by the HIF-1α inhibitor KC7F2. Four pooled PBMC samples were pretreated with 12.5 µM of KC7F2 for 30 minutes, followed by 24 hours stimulation with 100 ng/mL LPS (labeled by LK, orange), or a combination of LPS and DEX (LDK, lavender). Non-pretreated cells were also stimulated for 24 hours with either vehicle control (C, blue), 100 nM DEX (D, green), 100 ng/mL LPS (L, red), or a combination of LPS and DEX (LD, purple). Transcriptional responses of selected genes were measured using quantitative real-time PCR. Genes were grouped based on their functions into four subcategories: **(A)** Immune response genes. **(B)** Glycolysis-related genes. **(C)** TCA cycle-related genes. **(D)** Glucocorticoid response genes. The qPCR data were analyzed using ordinary one-way ANOVA followed by Sidak’s multiple comparisons test. The bars show mean + standard error of the mean. *p < 0.05, **p < 0.01, ***p < 0.001, and ****p < 0.0001.

Therefore, and to investigate the function of HIF-1α in more detail, we challenged the four PBMC pools for 24 hours with LPS, DEX, LPS+DEX, LPS+KC7F2, and a combination of all three agents LPS+DEX+KC7F2, and analyzed the mRNA expression of selected candidates from relevant immune, metabolic, and signaling pathways using qPCR. The expression profiles of individual genes and the results of the statistical analysis are presented in **Figure 7** and **Supplementary Table S5**, respectively. With the exception of *LDHA*, *EGLN1*, and *PDHA1*, the overall treatment effect was significant for all other genes. Pro-inflammatory cytokines (*IL1B*, *TNFA*) and canonical LPS-response genes (*SOD2*, *ACOD1*) showed the expected significant upregulation by LPS, which was for most of them (except *SOD2*) effectively suppressed by co-treatment with DEX (**Figure 7A**). Surprisingly, considering the reduced production of TNF-α by KC7F2 pretreatment in the previous experiment (paragraph 3.3), KC7F2 significantly enhanced the LPS-induced expression of *TNFA*, and non-significantly increased *IL1B* (**Figure 7A**). The induction of *ACOD1* by LPS, a potential target of HIF-1α (27), was decreased by KC7F2 approximately twofold (**Figure 7A**), but this effect did not reach statistical significance. On the other hand, KC7F2 effectively counteracted the suppressive effect of DEX in this group of genes (comparison between LPS+DEX vs. LPS+DEX+KC7F2; **Supplementary Table S5**). We measured the expression of *RELB* (encoding the RelB subunit of NF-κB) to examine if NF-κB, a central transcription factor in the inflammatory response, may be responsible for the observed expression changes. The profile of *RELB* was indeed similar and highly correlated to that of the pro-inflammatory cytokines (**Figure 7A**), particularly *TNFA* (**Supplementary Table S6**). In addition, we also measured the expression of *NFE2L2* encoding NRF2 - a transcription factor activated by itaconate. Itaconate is a metabolite produced by aconitate decarboxylase 1 (encoded by *ACOD1)* with anti-inflammatory properties mediated partly by NRF2 (28). However, the profile of *NFE2L2* differs from *ACOD1*, and appears to be dependent on HIF-1α, as it was increased by KC7F2 (**Figure 7A**).

The analyzed glycolysis-related genes each showed a distinct response pattern (**Figure 7B**). Whereas the profile of the glucose transporter *SLC2A1* (encoding GLUT1) was similar to *TNFA*, the profile of *SLC2A3* (encoding GLUT3) was more similar to *ACOD1*, which is also reflected by the correlation analysis (**Supplementary Table S6**). Overall, *SLC2A3* featured higher abundance and larger fold changes between treatments than *SLC2A1*, but only the LPS-induced upregulation reached statistical significance. *PFKFB3*, encoding an enzyme (6-phosphofructo-2-kinase/fructose-2,6-biphosphatase 3) promoting glycolytic flux and pro-inflammatory switch (8), was significantly upregulated by the LPS treatment, but the effect of LPS does not appear to be influenced by KC7F2. Unlike LPS, the effect of DEX was context-dependent; DEX alone tended to upregulate the expression of *PFKFB3*, but blunted its induction by LPS, which in turn was abolished by KC7F2 addition.

The expression profile of the two TCA cycle-related genes *IDH1* and *SDHB* (**Figure 7C**) indicates the widely described break in the TCA cycle caused by LPS (26). DEX blocked the LPS-induced downregulation of *IDH1* as well as *SDHB*, but this effect was offset by KC7F2. However, the differences between the treatments only reached statistical significance for *SDHB* (**Supplementary Table S5**). Interestingly, the transcriptional profiles of *IDH1* and *SDHB* appear to be inversely related to that of *TNFA* and *RELB*, as evidenced also by their strong negative correlation (**Supplementary Table S6**). The accumulation of citrate, caused by the break of the TCA cycle at isocitrate dehydrogenase, is coupled with itaconate production. Itaconate inhibits succinate dehydrogenase and thus contributes to succinate accumulation, which in turn promotes HIF-1α production (28). Despite the changes in expression of metabolic genes indicating altered activity of HIF-1α, none of the treatments significantly influenced mRNA expression of *HIF1A* and of *EGLN1* encoding the prolyl hydroxylase PHD2, which is regulated, i.a., by citrate and succinate accumulation, and destabilizes HIF-1α. However, their profiles indicate a potential feedback regulation of HIF-1α expression at the mRNA level.

There is a bidirectional crosstalk between HIF-1α and GR signaling (29). Indeed, Stifel et al. (19) showed stronger induction of two anti-inflammatory targets of GR - *GILZ* (official designation is *TSC22D3*, but *GILZ* is commonly used) and *DUSP1* by DEX in LPS-stimulated macrophages lacking HIF-1α compared to wild-type cells. In line with this finding, we observed enhanced upregulation of *GILZ* and *DUSP1* by KC7F2 when the PBMCs were treated with DEX in the presence of LPS compared to DEX alone (**Figure 7D**). However, KC7F2 actually increased *GILZ* and *DUSP1* expression not only in the presence of DEX (and LPS) but also when stimulated by LPS without DEX. The expression of *DUSP1* was also induced by LPS treatment alone. In contrast, induction of the canonical GR target *FKBP5* by DEX tended to be reduced by KC7F2. Likewise, the mRNA expression of the glucocorticoid receptor (referred to as *GR*, official designation is *NR3C1*), which was downregulated by DEX, tended to be increased by KC7F2. These results suggest, on the one hand, that KC7F2 in fact dampened GR signaling, which would explain the consistent loss of efficacy of DEX in this treatment. On the other hand, this also suggests that the upregulation of *GILZ* and *DUSP1* by KC7F2 occurs by a GR-independent mechanism. In an attempt to uncover this mechanism, we measured the mRNA expression of two potential *DUSP1* and *GILZ* regulators, *IL10* and *IRAK3*, which may be influenced by HIF-1α modulation (11, 30, 31). Whereas the expression profile of *IRAK3* was distinct from that of *GILZ* and *DUSP1*, the profile of *IL10* showed some similarity (**Figure 7A**), particularly with *DUSP1*, as reflected also by the correlation analysis (**Supplementary Table S6**). Thus, *IL10* could be involved, at least partly, in the GR-independent upregulation of *GILZ* and *DUSP1*.

**Figure 8:**
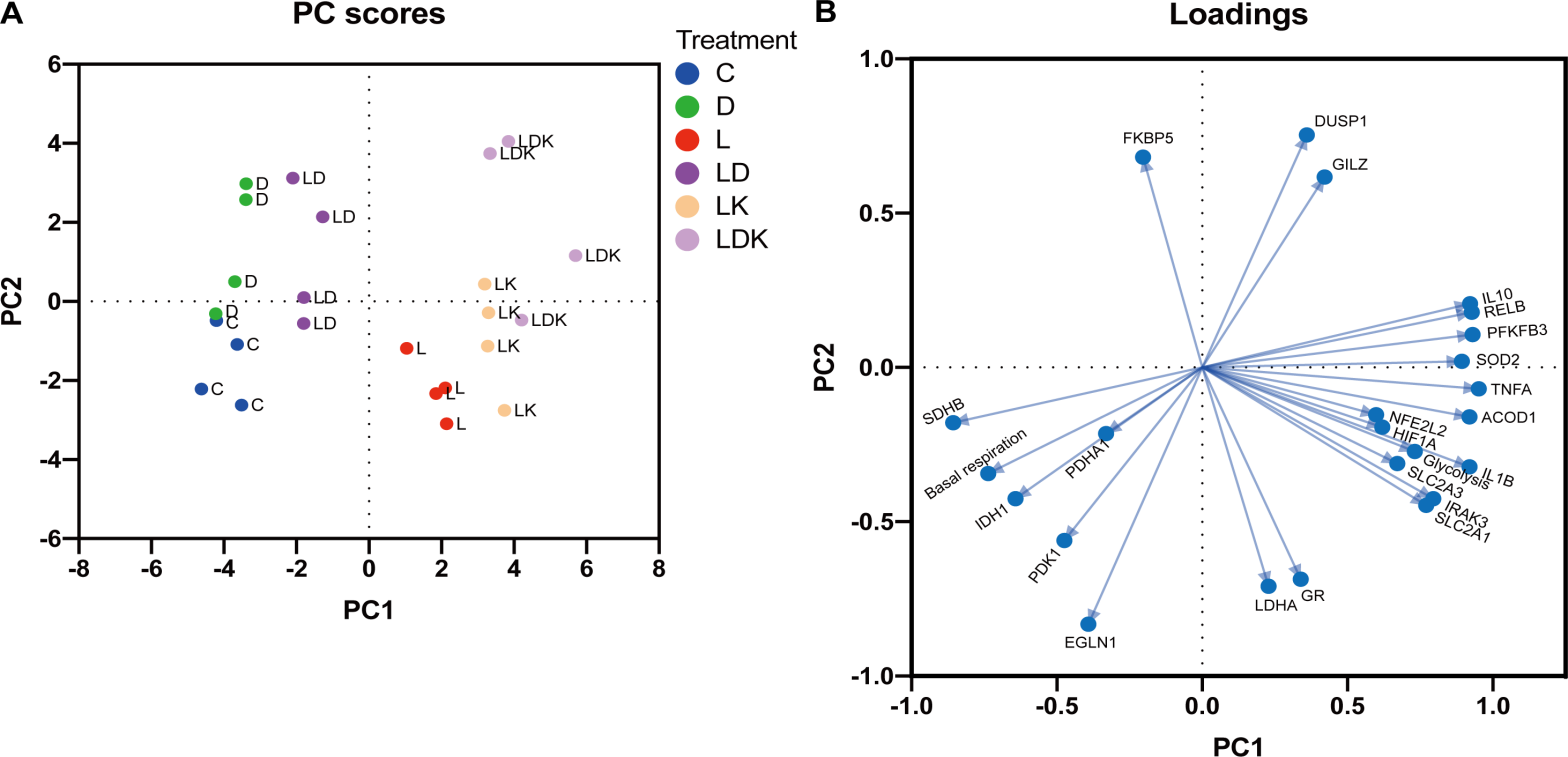
Principal component analysis (PCA) of the responses of PBMC to LPS, DEX and KC7F2 shows the relationship between the immune and metabolic status. **(A)** PC Scores Plot: The PCA scores plot reveals segregation of the PBMC samples along the first two principal components (PC1 and PC2) depending on treatment. Treatments are color-coded: Control (labeled by C, blue), DEX (D, green), LPS (L, red), LPS + DEX (LD, purple), LPS + KC7F2 (LK, orange), and LPS + DEX + KC7F2 (LDK, lavender). Overall, PC1 separates samples based on their inflammatory response, and PC2 based on their response to DEX. **(B)** Loading Plot: The loading plot displays the contribution of individual variables (genes or metabolic parameters) to the principal components. The length and direction of the arrows indicate the strength and direction of the relationship.

In order to get an overall picture of the relationships between the different functional groups of genes and the metabolic flux, we performed a principal component analysis in addition to the correlation analysis. As shown in **Figure 8**, the first two principal components separate the treatment groups quite well (**Figure 8A**). The first principal component (PC1), characterized by high positive loading of features associated with inflammation and glycolysis on the one side, and high negative loading of those representing TCA cycle and respiration on the opposite side, clearly demonstrates the connection between the immune and metabolic state of the cells (**Figure 8B**). The treatment groups containing KC7F2 show the strongest rightward shift (**Figure 8A**), emphasizing enhanced LPS-response by KC7F2. The second principal component (PC2) is defined by the response to DEX, and is outlined mainly by the canonical GR targets. The PCA plot (**Figure 8B**) further highlights the close connection between the glucose transporter *SLC2A3* with glycolysis and inflammation on the one hand, and of the TCA cycle genes *SDHB* and *IDH1* with basal respiration and resting state on the other.

## 4 Discussion

In the present study, we investigated for the first time the nexus between the inflammatory response and energy metabolism in porcine immune cells. Overall, our findings revealed similar fundamental relationships previously described in humans and rodents (2), but also notable differences. These could be due to the differences between species, but cell-specific differences likely also play an important role. Here, we have used PBMCs in a first attempt to discover sources of individual variation, but in the settings of our study neither sex nor husbandry conditions showed detectable effects. In addition to their suitability for diagnostic purposes, PBMCs also preserve some functionally relevant interactions between blood immune cells and can therefore mimic the *in vivo* situation more closely than isolated cell types (12). On the other hand, the mixed cell population of PBMCs complicates elucidating the underlying mechanisms. This may explain why we found responses in the bulk transcriptome data at the gene level, which may be those shared between cell types, rather than on the pathway level, which may be obscured by cell-specific differences.

Our results demonstrate the well-established tight connection between the LPS-induced pro-inflammatory response and a shift in cell metabolism towards oxidative glycolysis (4). However, we found no evidence for impaired mitochondrial respiration and ATP production as occurs in inflammatory murine macrophages or activated T cells due to the inhibition of the mitochondrial electron transport chain (ETC) by nitric oxide (12, 32). Similar to human macrophages, the explanation for this difference could lie in the very low abundance of *NOS2*, encoding the inducible nitric oxide synthase, in porcine PBMCs. Actually, the Mito Stress Test evidenced that LPS significantly enhanced the spare respiratory capacity of porcine PBMCs, which is a striking observation considering the evidence from our expression analysis that the TCA cycle is impaired by LPS at IDH and SDH. Our data show that parallel to this perturbation, LPS tended to downregulate the expression of *PDK1* encoding the pyruvate dehydrogenase kinase. This could compensate for the deficiency in TCA cycle by allowing more pyruvate to be converted to acetyl-CoA and enter the cycle. Furthermore, LPS increased expression of the glucose transporter genes, particularly *SLC2A3* (about 6-fold compared to about 1.5 for *SLC2A1*), which could help to maintain supply for mitochondrial respiration despite increased aerobic glycolysis demonstrated by Glycolysis Stress Test. In fact, a recent study in Th17 cells evidenced that GLUT3-dependent glucose uptake fuels not only glycolysis but also mitochondrial respiration (9). Yet, other important metabolic pathways are also involved in the metabolic reprogramming of activated immune cells, such as fatty acid oxidation (32), which we did not cover in our study. Preservation of mitochondrial respiration in porcine PBMCs may be important for lymphocyte function, as demonstrated for T-cell activation (33) or humoral immunity in B-cells (34), but appears dispensable for the cytokine response, as suggested by the lack of influence of oligomycin pretreatment on TNF-α production. Indeed, a recent study suggested that the accumulation of TCA cycle-derived immunometabolites can occur without respiratory inhibition (14). In contrast to LPS, dexamethasone reduced both glycolysis as well as mitochondrial respiration, and in the combined treatment counteracted the metabolic actions of LPS. The fact that dexamethasone influences metabolism of immune cells and how this relates to its anti-inflammatory action remained unexplored until recently. Two studies reported an effect of glucocorticoids on the TCA cycle in LPS-stimulated murine bone marrow-derived macrophages (mBMDM). Auger et al. (35) suggested that glucocorticoids (dexamethasone) trigger an accelerated flux of the TCA cycle that facilitates enhanced production of the anti-inflammatory metabolite itaconate and that this mechanism accounts for a substantial part of their anti-inflammatory effects. However, our data clearly show that dexamethasone effectively suppressed the LPS-induced upregulation of *ACOD1* expression, arguing against the mechanism proposed by Auger et al. (35) in porcine PBMCs. Stifel et al. (19) also found that dexamethasone promotes the TCA cycle flux, but they discovered that this prevents succinate accumulation and concluded that coordination of succinate metabolism is an important part of the anti-inflammatory effects of glucocorticoids. Consistent with the findings of the two studies in mBMDM, our results evidence that the LPS-induced perturbation of the TCA cycle is abolished by dexamethasone. Whether this prevents succinate and/or citrate accumulation, and how this influences the overall profile of TCA cycle-derived immunometabolites and ultimately relates to the anti-inflammatory action of glucocorticoids in porcine PBMCs warrants further investigation. It is astonishing that despite maintaining baseline expression of the TCA cycle genes, including *SDHB* which is not only a part of the TCA cycle but also of the ETC, dexamethasone consistently lowered mitochondrial respiration. This is yet another striking contradiction compared to mBMDM (19, 35) and also to the widely accepted notion that oxidative metabolism is associated with a resting or reparatory (also called alternatively activated) state of immune cells (26). It remains to be investigated whether such a low energetic state induced by dexamethasone, and glucocorticoids in general, could impair the response to a subsequent immune stimulus, especially during chronic stimulation (36). While we cannot rule out involvement of other metabolic pathways, we hypothesize that the low energetic state could be caused, at least partly, by reduced supply of glucose due to downregulation of the glucose transporter genes by dexamethasone. In addition to the mechanisms centered around the effect of dexamethasone on the TCA cycle postulated by Auger et al. (35) and Stifel et al. (19), Clayton et al. (20) suggested that the anti-inflammatory effect of dexamethasone in macrophages is based on inhibition of HIF1α-dependent glucose uptake via down-regulation of GLUT1 expression. So far, research on the role of glucose transporters in immunometabolism, specifically the link between pro-inflammatory state and glycolytic metabolism, concentrated mainly on GLUT1 (37). Our results, together with the findings of Hochrein et al. (9) in Th17 cells, suggest that GLUT3 *(SLC2A3*) likely plays a more important role in the antagonistic immunometabolic action of LPS and dexamethasone than GLUT1. As demonstrated by our mRNA-seq results, unlike *SLC2A1*, *SLC2A3* expression is regulated by LPS and DEX already after 2 hours of stimulation similar to pro-inflammatory genes such as *TNFA*, and importantly, *HIF1A*. Moreover, the transcriptional response of *SLC2A3* corresponds with changes in the glycolytic metabolism of PBMCs induced by LPS, DEX, and KC7F2. In particular, this could explain the downregulation of both glycolysis and mitochondrial respiration by dexamethasone applied alone and the apparently paradoxical enhancement of glycolysis by KC7F2 in the presence of dexamethasone. The contrasting transcriptional responses of *SLC2A1* and *SLC2A3* to LPS and dexamethasone in the presence of KC7F2 add further evidence for different mechanisms of their transcriptional regulation and possibly distinct functions. The similar expression profiles of *SLC2A3* and *ACOD1* indicate that GLUT3 may influence the production of TCA-derived immunometabolites. Whereas the regulation of *SLC2A3* could explain the metabolic actions of dexamethasone, it is not consistent with the decline in mitochondrial respiration induced by KC7F2. This effect of KC7F2 was surprising, as inhibition of HIF-1α translation was anticipated to promote a switch from glycolytic to oxidative metabolism (21), but both were reduced. A possible mechanism for the reduction in mitochondrial respiration caused by KC7F2 could be the upregulation of *GILZ*, because a recent study reported decreased mitochondrial respiration in macrophages overexpressing GILZ (38). Another striking observation was that, overall, KC7F2 enhanced the transcriptional response to LPS and blunted the response to dexamethasone. Most surprisingly, the *TNFA* response was also enhanced despite the reduced TNF-α production indicated by the immunoassay. It is important to note that the immunoassay results reflect the accumulation of TNF-α in the cell culture media during the 24 hour stimulation. The LPS response is regulated in a strictly time-dependent manner; TNF-α production reaches its peak in the early phase and declines by an order of magnitude after 24 hours of stimulation. It is assumed that the effect of HIF-1α is also time-dependent, whereby it initially triggers inflammation and later, when permanently activated, dampens it (11). Consequently, most of TNF-α likely accumulated in the media in the early phase, when inhibition of HIF-1α is expected to curb inflammation. Notably, the expression of canonical GR targets (e.g. *FKBP5*) is considerably less affected by KC7F2 than the expression of the metabolic and immune response genes. In this context, the strong antagonistic correlation between the TCA genes (*IDH1* and *SDHB*) and NF-κB signaling pathway genes (e.g. *RELB* or *TNFA*) is particularly noteworthy. The TCA-derived immunometabolites, including itaconate, citrate, and succinate, are involved in epigenetic regulation of gene expression, i.a., via changes in DNA methylation mediated by the ten-eleven translocation family member 2 (TET2) and post-translational modification of proteins (itaconation, acetylation, and succinylation, respectively (39), including histone acetylation, and thus chromatin accessibility (9). Consequently, the observed effects of KC7F2 indicate that the crosstalk between HIF-1α and GR signaling likely includes indirect interactions via epigenetic and post-translational mechanisms. Interestingly, acetylation of GR (40) as well as of NF-κB (41) has been shown to influence their transcriptional activity. Moreover, Hochrein et al. (9) demonstrated that GLUT3 promotes the expression of inflammatory cytokines in Th17 cells via acetyl-CoA generation and locus-specific histone acetylation, providing an additional potential link between metabolic targets of GR and the chromatin landscape.

In summary, our study revealed novel, fundamental insights into the metabolic rewiring of immune cells by LPS and glucocorticoids. Our findings provide strong evidence that GLUT3, rather than GLUT1, determines the glycolytic metabolism of porcine PBMCs. Furthermore, while previous studies concentrated largely on the role of succinate metabolism in the immunometabolic action of DEX, our results show that the regulation of isocitrate dehydrogenase likely plays an important role as well. Shaping the epigenetic landscape by controlling the production of immunometabolites could be another component of the GR’s repertoire of regulatory mechanisms. More research is required to confirm and fully understand the role of *SLC2A3*, *IDH1*, and *SDHB* in mediating the anti-inflammatory action of GR. Although due to species differences caution is advised, our findings may also inform future studies of immunometabolism and the role of glucocorticoids in this process in humans.

## Data Availability

Publicly available datasets were analyzed in this study. This data can be found here: https://www.ebi.ac.uk/biostudies/arrayexpress/studies/E-MTAB-9808.

## References

[1] Hortova-Kohoutkova M Laznickova P Fric J. How immune-cell fate and function are determined by metabolic pathway choice: The bioenergetics underlying the immune response. BioeEssays. (2021) 43:e2000067. doi: 10.1002/bies.202000067 33191545

[2] Verberk SGS de Goede KE Gorki FS van Dierendonck X Arguello RJ Van den Bossche J. An integrated toolbox to profile macrophage immunometabolism. Cell Rep Methods. (2022) 2:100192. doi: 10.1016/j.crmeth.2022.100192 35497494 PMC9046227

[3] Voss K Hong HS Bader JE Sugiura A Lyssiotis CA Rathmell JC. A guide to interrogating immunometabolism. Nat Rev Immunol. (2021) 21:637–52. doi: 10.1038/s41577-021-00529-8 PMC8478710 33859379

[4] O’Neill LA Kishton RJ Rathmell J. A guide to immunometabolism for immunologists. Nat Rev Immunol. (2016) 16:553–65. doi: 10.1038/nri.2016.70 PMC5001910 27396447

[5] Palmer CS Ostrowski M Balderson B Christian N Crowe SM. Glucose metabolism regulates T cell activation, differentiation, and functions. Front Immunol. (2015) 6:1. doi: 10.3389/fimmu.2015.00001 25657648 PMC4302982

[6] Mills EL Kelly B Logan A Costa AS Varma M Bryant CE. Succinate dehydrogenase supports metabolic repurposing of mitochondria to drive inflammatory macrophages. Cell. (2016) 167:457–70.e13. doi: 10.1016/j.cell.2016.08.064 27667687 PMC5863951

[7] Tannahill G Curtis A Adamik J Palsson-McDermott E McGettrick A Goel G. Succinate is an inflammatory signal that induces IL-1β through HIF-1α. Nature. (2013) 496:238–42. doi: 10.1038/nature11986 PMC4031686 23535595

[8] Rodríguez-Prados J-C Través PG Cuenca J Rico D Aragonés J Martin-Sanz P. Substrate fate in activated macrophages: a comparison between innate, classic, and alternative activation. J Immunol. (2010) 185:605–14. doi: 10.4049/jimmunol.0901698 20498354

[9] Hochrein SM Wu H Eckstein M Arrigoni L Herman JS Schumacher F. The glucose transporter GLUT3 controls T helper 17 cell responses through glycolytic-epigenetic reprogramming. Cell Metab. (2022) 34:516–32.e11. doi: 10.1016/j.cmet.2022.02.015 35316657 PMC9019065

[10] Jha AK Huang SC-C Sergushichev A Lampropoulou V Ivanova Y Loginicheva E. Network integration of parallel metabolic and transcriptional data reveals metabolic modules that regulate macrophage polarization. Immunity. (2015) 42:419–30. doi: 10.1016/j.immuni.2015.02.005 25786174

[11] Shalova IN Lim JY Chittezhath M Zinkernagel AS Beasley F Hernández-Jiménez E. Human monocytes undergo functional re-programming during sepsis mediated by hypoxia-inducible factor-1α. Immunity. (2015) 42:484–98. doi: 10.1016/j.immuni.2015.02.001 25746953

[12] Karan KR Trumpff C McGill MA Thomas JE Sturm G Lauriola V. Mitochondrial respiratory capacity modulates LPS-induced inflammatory signatures in human blood. BBI-Health. (2020) 5:100080. doi: 10.1016/j.bbih.2020.100080 33073254 PMC7561023

[13] Jones N Piasecka J Bryant AH Jones R Skibinski D Francis NJ. Bioenergetic analysis of human peripheral blood mononuclear cells. Clin Exp Immunol. (2015) 182:69–80. doi: 10.1111/cei.12662 26032049 PMC4578510

[14] Ball AB Jones AE Nguyễn KB Rios A Marx N Hsieh WY. Pro-inflammatory macrophage activation does not require inhibition of mitochondrial respiration. bioRxiv. (2024). doi: 10.1101/2024.05.10.593451 PMC11850891 39753784

[15] Meyer MM Lamont SJ Bobeck EA. Mitochondrial and glycolytic capacity of peripheral blood mononuclear cells isolated from diverse poultry genetic lines: optimization and assessment. Front Vet Sci. (2021) 8:815878. doi: 10.3389/fvets.2021.815878 35155649 PMC8831803

[16] Adler M Murani E Ponsuksili S Wimmers K. PBMC transcription profiles of pigs with divergent humoral immune responses and lean growth performance. Int J Biol Sci. (2013) 9:907–16. doi: 10.7150/ijbs.6769 PMC3805897 24155665

[17] Li Z Hadlich F Wimmers K Murani E. Glucocorticoid receptor hypersensitivity enhances inflammatory signaling and inhibits cell cycle progression in porcine PBMCs. Front Immunol. (2022) 13:976454. doi: 10.3389/fimmu.2022.976454 36505401 PMC9730246

[18] Timmermans S Souffriau J Libert C. A general introduction to glucocorticoid biology. Front Immunol. (2019) 10:1545. doi: 10.3389/fimmu.2019.01545 31333672 PMC6621919

[19] Stifel U Wolfschmitt E-M Vogt J Wachter U Vettorazzi S Tews D. Glucocorticoids coordinate macrophage metabolism through the regulation of the tricarboxylic acid cycle. Mol Metab. (2022) 57:101424. doi: 10.1016/j.molmet.2021.101424 34954109 PMC8783148

[20] Clayton SA Lockwood C O’Neil JD Daley KK Hain S Abdelmottaleb D. The glucocorticoid dexamethasone inhibits HIF-1α stabilization and metabolic reprogramming in lipopolysaccharide-stimulated primary macrophages. Discovery Immunol. (2023) 2:kyad027. doi: 10.1093/discim/kyad027 PMC10917182 38567068

[21] McGettrick AF O’Neill LA. The role of HIF in immunity and inflammation. Cell Metab. (2020) 32:524–36. doi: 10.1016/j.cmet.2020.08.002 32853548

[22] Muráni E Ponsuksili S Jaeger A Görres A Tuchscherer A Wimmers K. A naturally hypersensitive glucocorticoid receptor elicits a compensatory reduction of hypothalamus–pituitary–adrenal axis activity early in ontogeny. Open Biol. (2016) 6:150193. doi: 10.1098/rsob.150193 27440422 PMC4967818

[23] Brückmann R Tuchscherer M Tuchscherer A Gimsa U Kanitz E. Early-life maternal deprivation predicts stronger sickness behaviour and reduced immune responses to acute endotoxaemia in a pig model. Int J Mol Sci. (2020) 21:5212. doi: 10.3390/ijms21155212 32717860 PMC7432595

[24] Murani E Trakooljul N Hadlich F Ponsuksili S Wimmers K. Transcriptome responses to dexamethasone depending on dose and glucocorticoid receptor sensitivity in the liver. Front Genet. (2019) 10:559. doi: 10.3389/fgene.2019.00559 31249595 PMC6582245

[25] Li Z Trakooljul N Hadlich F Ponsuksili S Wimmers K Murani E. Transcriptome analysis of porcine PBMCs reveals lipopolysaccharide-induced immunomodulatory responses and crosstalk of immune and glucocorticoid receptor signaling. Virulence. (2021) 12:1808–24. doi: 10.1080/21505594.2021.1948276 PMC8296968 34288827

[26] Van den Bossche J O’Neill LA Menon D. Macrophage immunometabolism: where are we (going)? Trends Immunol. (2017) 38:395–406. doi: 10.1016/j.it.2017.03.001 28396078

[27] Li Y Li Y-C Liu X-T Zhang L Chen Y-H Zhao Q. Blockage of citrate export prevents TCA cycle fragmentation via Irg1 inactivation. Cell Rep. (2022) 38. doi: 10.1016/j.celrep.2022.110391 35172156

[28] O’Neill LAJ Artyomov MN. Itaconate: the poster child of metabolic reprogramming in macrophage function. Nat Rev Immunol. (2019) 19:273–81. doi: 10.1038/s41577-019-0128-5 30705422

[29] Vanderhaeghen T Beyaert R Libert C. Bidirectional crosstalk between hypoxia inducible factors and glucocorticoid signalling in health and disease. Front Immunol. (2021) 12:684085. doi: 10.3389/fimmu.2021.684085 34149725 PMC8211996

[30] Su J Xie Q Wilson I Li L. Differential regulation and role of interleukin-1 receptor associated kinase-M in innate immunity signaling. Cell Signal. (2007) 19:1596–601. doi: 10.1016/j.cellsig.2007.02.009 PMC1978187 17379480

[31] Berrebi D Bruscoli S Cohen N Foussat A Migliorati G Bouchet-Delbos L. Synthesis of glucocorticoid-induced leucine zipper (GILZ) by macrophages: an anti-inflammatory and immunosuppressive mechanism shared by glucocorticoids and IL-10. Blood. (2003) 101:729–38. doi: 10.1182/blood-2002-02-0538 12393603

[32] Wang R Green DR. Metabolic reprogramming and metabolic dependency in T cells. Immunol Rev. (2012) 249:14–26. doi: 10.1111/j.1600-065X.2012.01155.x 22889212 PMC3422760

[33] Sena LA Li S Jairaman A Prakriya M Ezponda T Hildeman DA. Mitochondria are required for antigen-specific T cell activation through reactive oxygen species signaling. Immunity. (2013) 38:225–36. doi: 10.1016/j.immuni.2012.10.020 PMC3582741 23415911

[34] Urbanczyk S Baris OR Hofmann J Taudte RV Guegen N Golombek F. Mitochondrial respiration in B lymphocytes is essential for humoral immunity by controlling the flux of the TCA cycle. Cell Rep. (2022) 39. doi: 10.1016/j.celrep.2022.110912 35675769

[35] Auger J-P Zimmermann M Faas M Stifel U Chambers D Krishnacoumar B. Metabolic rewiring promotes anti-inflammatory effects of glucocorticoids. Nature. (2024) 629:184–92. doi: 10.1038/s41586-024-07282-7 38600378

[36] Cribioli E Giordano Attianese GMP Ginefra P Signorino-Gelo A Vuillefroy de Silly R Vannini N. Enforcing GLUT3 expression in CD8+ T cells improves fitness and tumor control by promoting glucose uptake and energy storage. Front Immunol. (2022) 13:976628. doi: 10.3389/fimmu.2022.976628 36203587 PMC9530831

[37] Freemerman AJ Johnson AR Sacks GN Milner JJ Kirk EL Troester MA. Metabolic reprogramming of macrophages: glucose transporter 1 (GLUT1)-mediated glucose metabolism drives a proinflammatory phenotype. J Biol Chem. (2014) 289:7884–96. doi: 10.1074/jbc.M113.522037 PMC3953299 24492615

[38] Legroux TM Schymik HS Gasparoni G Mohammadi S Walter J Libert C. Immunomodulation by glucocorticoid-induced leucine zipper in macrophages: enhanced phagocytosis, protection from pyroptosis, and altered mitochondrial function. Front Immunol. (2024) 15:1396827. doi: 10.3389/fimmu.2024.1396827 38855102 PMC11157436

[39] Ryan DG Peace CG Hooftman A. Basic mechanisms of Immunometabolites in shaping the immune response. J Innate Immun. (2023) 15:925–43. doi: 10.1159/000535452 PMC10730108 37995666

[40] Ito K Yamamura S Essilfie-Quaye S Cosio B Ito M Barnes PJ. Histone deacetylase 2–mediated deacetylation of the glucocorticoid receptor enables NF-κB suppression. J Exp Med. (2006) 203:7–13. doi: 10.1084/jem.20050466 16380507 PMC2118081

[41] Santarsiero A Convertini P Todisco S Pierri CL De Grassi A Williams NC. ACLY nuclear translocation in human macrophages drives proinflammatory gene expression by NF-κB acetylation. Cells. (2021) 10:2962. doi: 10.3390/cells10112962 34831186 PMC8616537

