# Supplementary material for: Dexamethasone has profound influence on the energy metabolism of porcine blood leukocytes and prevents the LPS-induced glycolytic switch
Source: Front Immunol. 2025 Feb 25;16:1514061. doi: 10.3389/fimmu.2025.1514061 (PMC11893826; doi:10.3389/fimmu.2025.1514061)
Supplement: Supplementary file 7 [file DataSheet1.docx]

Supplementary Material

**Dexamethasone has profound influence on the energy metabolism of porcine blood leukocytes and prevents the LPS-induced glycolytic switch**

**Wenjuan Ma^1^, Julia Brenmoehl^2^, Nares Trakooljul^1^, Klaus Wimmers^1^, Eduard Murani^1^***

^1^Working Group Physiological Genomics, Research Institute for Farm Animal Biology (FBN), Dummerstorf, Germany

^2^Working Group Endocrinology of Farm Animals, Research Institute for Farm Animal Biology (FBN), Dummerstorf, Germany

* Correspondence:

Eduard Murani

murani@fbn-dummerstorf.de

# Supplementary Description of Husbandry Conditions

**Farrowing TW1:**

In the free access stall the sows and piglets were provided with approx. 6 m² space. The pens are equipped with plastic slatted floors, cast-iron elements in the sow's resting area and a piglet nest heated with hot water (0.75 m²) with climate cover. In addition, a heat lamp is used above the piglet nest during the first days of the piglets' life. The heating and ventilation of the entire room is controlled by a climate computer. The sows are hand-fed twice daily on an animal-by-animal basis as needed (gradually increasing the daily feed amount from 1 kg after birth to 7-8 kg 14 days postpartum) with a complete feed for lactating sows. Piglets are offered a prestarter via a supplementary feeder from the 2nd week of life. Water was available to the animals ad libitum at all times. The suckling period lasted 4-weeks.

**Farrowing TW3:**

In the farrowing pens the sows and piglets were provided with approx. 8.4 m² space and free access to an outdoor run with 5 m². The pens had straw bedding. The sows were offered a jute cloth for occupation/nest building. For the piglets, a piglet nest with floor heating and heat lamp is integrated into the pen. The sows and piglets could move freely. The sows were fed a lactation feed according to the recommendations for energy and nutrient supply with the greatest possible avoidance of soy several times a day as required. Water was available to the animals ad libitum at all times. The suckling period lasted 6-weeks.

**Weaning TW1:**

After the 4-week suckling period, the piglets were grouped by body weight and sex into groups of max. 12 animals per (4.5 m²). The weaner pens were heated and ventilated by a climate computer according to a stored temperature curve. The floor consists of 2/3 plastic slatted floors and 1/3 heated thermal panels. In the first week the piglets were restrictively fed with a piglet rearing feed I. Afterwards they received a transition feed until approx. 45 days, and subsequently piglet rearing feed II ad libitum. Water was available to the animals ad libitum.

**Weaning TW3:**

After 6- weeks of suckling, entire litters were moved into weaner pens (7 m²) with free access to an outdoor run with 4.2 m², all bedded with straw. In order to meet the increased heat requirements of the animals, part of the pen is equipped with a variable climate cover in addition to the underfloor heating. The animals were fed with GMO-free piglet feed ad libitum via feed stations. Water was available to the animals ad libitum.

**Fattening TW1:**

At about 25 kg live weight, the piglets were transferred to fattening pens. Each fattening pen (approx. 12.1 m²) accommodated max. 10 animals. The pens are divided into a 6 m² paved lying area with bedding and a walking area with a concrete slatted floor, in which drinking troughs are also located. The animals were fed ad libitum with a pre- and final fattening feed via retrieval feeding stations according to their needs.

**Fattening TW3:**

Around the 70th day of life, the runners were moved to the fattening pens. Each fattening pen (approx. 15.3 m²) accommodated max. 10 animals. The fattening pens had access to an outdoor run with 12.4 m² space. The pens and runs were bedded with straw. The animals were fed with a pre-fattening and final fattening feed according to their needs and ad libitum via fattening stations. Water was available to the animals ad libitum.

# Supplementary Figures and Tables

## Supplementary Figures


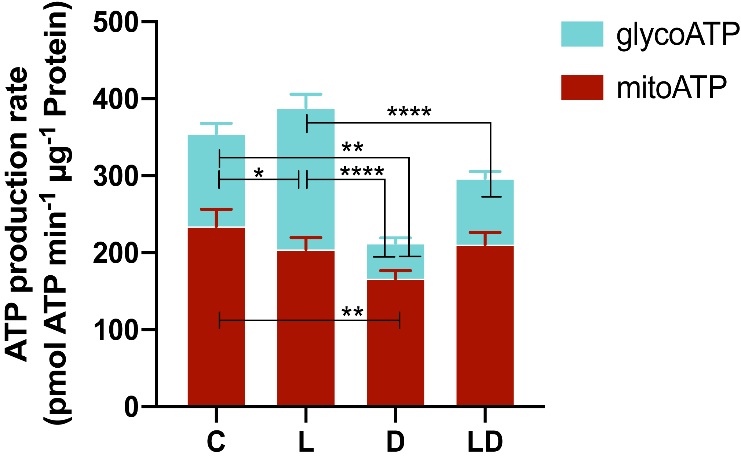


**Supplementary Figure 1. Different contribution from glycolytic and mitochondrial pathways to ATP production under various treatment conditions.** The ATP production rate (pmol ATP min⁻¹ µg⁻¹ protein) partitioned between glycolytic ATP (glycoATP, cyan) and mitochondrial ATP (mitoATP, red) across different treatment groups: vehicle control (C), LPS (L), DEX (D), and LPS + DEX (LD). The total height of each bar represents the sum of ATP production from both pathways. Data are presented as mean + standard error of the mean. Statistical significance was assessed using two-way ANOVA followed by Tukey´s multiple comparison test. *p < 0.05, **p < 0.01, ****p < 0.0001.


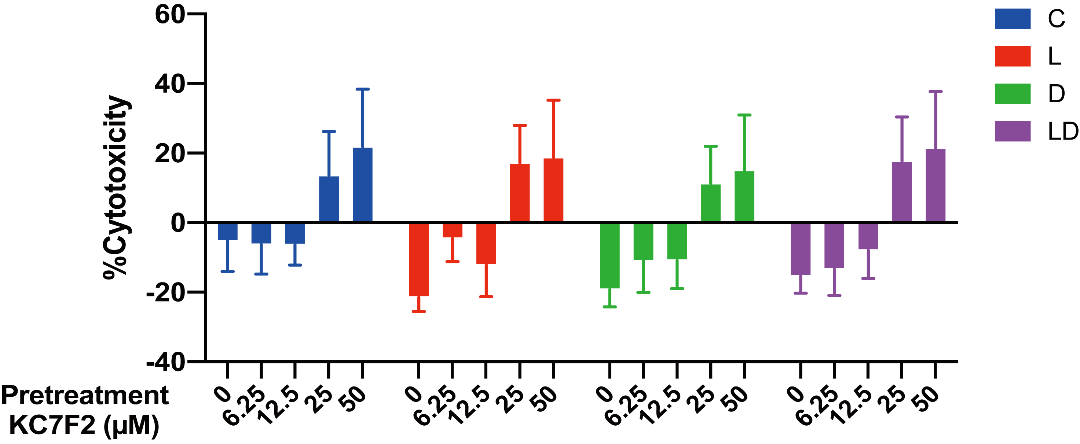


**Supplementary Figure 2.** **Cytotoxicity of KC7F2 in LPS and DEX treated PBMC.** Cytotoxicity of different concentrations of KC7F2 (0, 6.25, 12.5, 25, and 50 µM), followed by treatment with either vehicle control (C, blue), LPS (L, red), DEX (D, green), or a combination of LPS and DEX (LD, purple) was assessed in four pooled PBMC samples using the CyQUANT LDH Cytotoxicity assay. The cytotoxicity of the different conditions is expressed as a percentage change compared to the control, with positive values indicating increased cytotoxicity and negative values indicating decreased cytotoxicity compared to the untreated control. Data are presented as mean + standard error of the mean.
